# The Potential for Circulating Tumor Cells in Pancreatic Cancer Management

**DOI:** 10.3389/fphys.2017.00381

**Published:** 2017-06-02

**Authors:** Michael Pimienta, Mouad Edderkaoui, Ruoxiang Wang, Stephen Pandol

**Affiliations:** ^1^University of California, San Diego School of MedicineLa Jolla, CA, United States; ^2^Cedars-Sinai Medical Center, Basic and Translational Pancreas ResearchLos Angeles, CA, United States

**Keywords:** circulating tumor cells, pancreatic cancer, pancreatic adenocarcinoma, metastasis, epithelial to mesenchymal transition, mesenchymal to epithelial transitions

## Abstract

Pancreatic cancer is one the most lethal malignancies. Only a small proportion of patients with this disease benefit from surgery. Chemotherapy provides only a transient benefit. Though much effort has gone into finding new ways for early diagnosis and treatment, average patient survival has only been improved in the order of months. Circulating tumor cells (CTCs) are shed from primary tumors, including pre-malignant phases. These cells possess information about the genomic characteristics of their tumor source *in situ*, and their detection and characterization holds potential in early cancer diagnosis, prognosis, and treatment. Liquid Biopsies present an alternative to tumor biopsy that are hard to sample. Below we summarize current methods of CTC detection, the current literature on CTCs in pancreatic cancer, and future perspectives.

## Introduction

Virtually all cancers have the potential to metastasize, and metastatic disease comes about from a series of events involving the interplay between primary tumor cells and their microenvironment. The end result is the dissemination and growth of tumor cells in new tissue environments. First described in the literature in the nineteenth century by British surgeon James Paget, metastatic disease largely remains an unsolved worldwide public health concern today—metastasis accounts for more than 90% of cancer-related deaths (Ashworth, 1869; Spano et al., 2012).

We now know that metastasis is an “extremely complex” multistep process. Tumor cells must advance through an invasion-metastasis cascade. In order to produce clinically detectable lesions, primary tumor cells need to progressively intravasate through the basal membrane into the systemic or lymphatic circulation, survive in the circulatory environment, adhere to vessel walls, extravasate into a foreign tissue site, and adapt, survive, and proliferate in their new microenvironment (Fidler, 2003).

**Figure 1:**
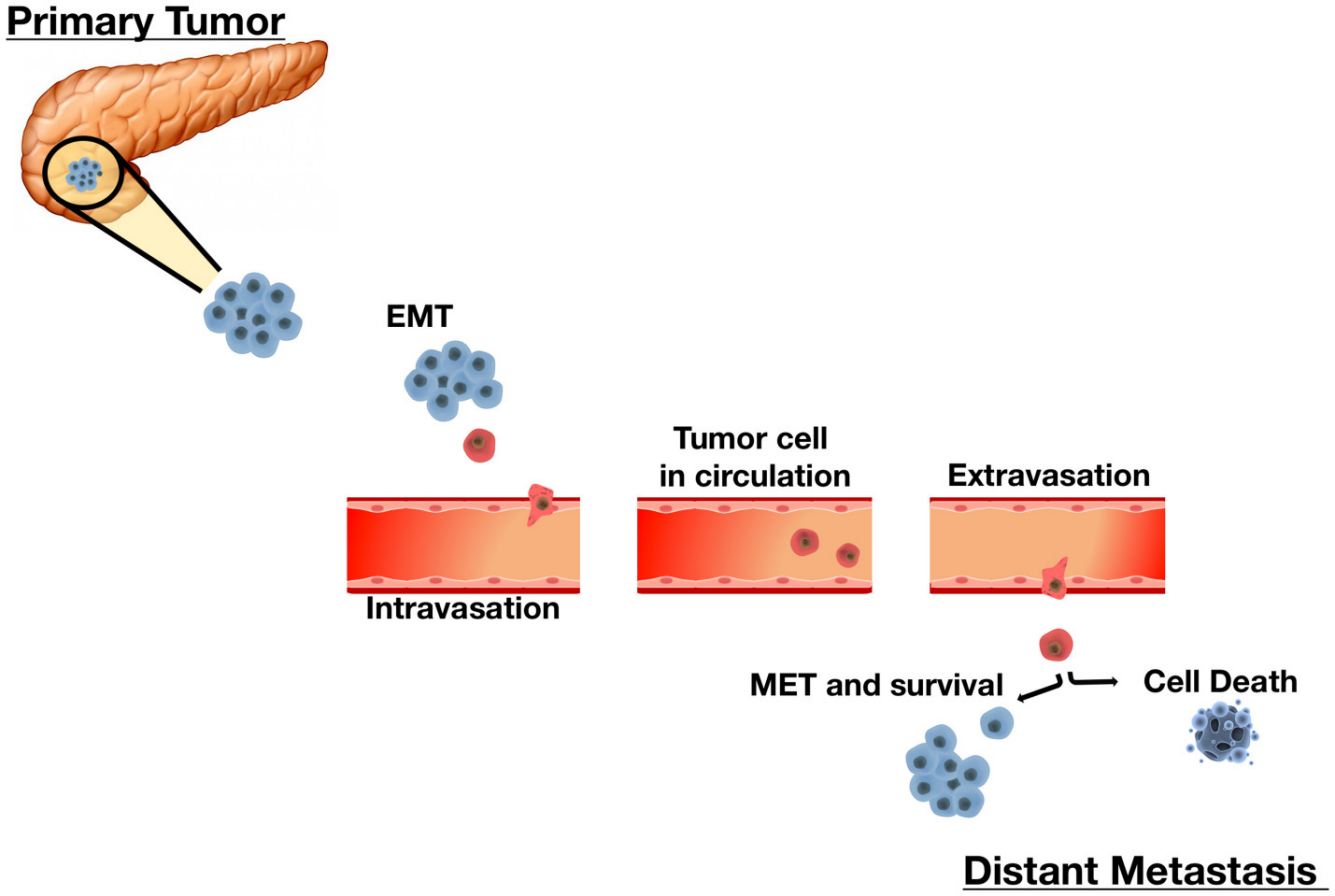
The Sequential Process of Cancer Metastasis. Metastasis is a complex multi-step process. Tumor cells undergo remarkable morphological and phenotypical changes enabling migration and infiltration of adjacent sites as single cells or small clusters. An initial epithelial-to-mesenchymal transition (EMT) allows cells to acquire mesenchymal properties essential for motility and migration. Upon infiltrating local stroma, cancer cells intravasate into the vascular or lymphatic system and circulate throughout the body as circulating tumor cells (CTCs). In the circulation, disseminated CTCs must overcome barriers such as sheer-stress and the immune system. CTCs that survive the circulation extravasate and invade distant tissues by reestablishing characteristics of their corresponding primary tumor. CTC plasticity allows them to undergo the mesenchymal-to-epithelial transition (MET) to achieve this. Each step in this metastasis cascade is rate-limiting. Cells that successfully adapt to their microenvironment and resume proliferation successfully form overt secondary tumors. Alternately, cells that do not survive undergo cell death.

The potential for mobilization and invasion are critical to the process of intravasation. Cells should be able to degrade the extracellular matrix (ECM) and secrete proteolytic enzymes to facilitate migration and intravasation into the circulatory system. These epithelium-derived cancer cells are thought to undergo a morphological change of epithelial-to-mesenchymal transition (EMT; Leber and Efferth, 2009; Dhamija and Diederichs, 2016). At metastatic sites, neoplastic cells should also infiltrate the endothelium in order to colonize new tissues and be able to induce neo-angiogenesis to ensure sufficient blood supply to the newly formed tumor in order to maintain metabolic needs (Leber and Efferth, 2009; Figure 1). The preferred growth and survival of cancer cells at certain metastatic sites is less understood, although a reverse behavioral change, mesenchymal-to-epithelium transition (MET), is hypothesized to take place for this to occur (Dhamija and Diederichs, 2016).

Metastasis itself is a highly inefficient process, as each step in the metastasis cascade may play a limiting role in disease progression, that is—if one fails then all fails. Only few cancer cells are able to go on to form malignant secondary tumors. Animal studies on the kinetics of each step have shown that post-extravasation steps create the largest barrier to metastasis. An early report, for example, found that 80% of injected cancer cells survive in circulation and extravasate into distant tissues, but only 1 in 40 cells formed micrometastasis and 1 in 100 micrometastasis actually progressed to macroscopic tumors (Luzzi et al., 1998). Further, studies found similar results, showing the high efficiency of extravasation and survival in the circulation to be independent of cell's malignant potential (Koop et al., 1995, 1996; Cameron et al., 2000). These findings suggest that the growth of circulating tumor cells (CTCs) in a new microenvironment is a key step in metastatic tumor formation.

Materials shed from tumors are being investigated for their potential use in diagnosis, prognosis, and management of cancer. CTCs, cell-free circulating tumor DNA (ctDNA), and tumor cell produced exosomes (oncosomes) all hold promise in the current cancer research for future clinical use in diagnosis and management. Oncosomes, nanovesicles actively shed from most types of cancers in large numbers, like CTCs, are a dynamic source of information regarding the genomic characteristics from the parent tumor of which they are release (Deneve et al., 2013; Pantel and Alix-Panabieres, 2013; Speicher and Pantel, 2014). Though each has their own technical challenges, these materials are now being isolated and detected in peripheral blood of patients with many types metastatic cancers and are leading the way toward use of “liquid biopsies.”

## CTCs in modern cancer research

While, it is estimated that only 0.01% of CTCs have metastatic potential, the clinical importance of CTCs in “modern cancer research” over the past two decades has become increasingly apparent (Zhe et al., 2011). Although the presence of CTCs in blood could not exclusively indicate clinically significant macro-metastases, partly due to metastatic inefficiency, it surely indicates the presence of malignant tumors *in situ*. More and more data suggests that solid tumor shedding occurs early in disease. A recent study detected disseminated tumor cells (DTCs) in bone marrow of a mouse breast cancer model during pre-malignant stages, reinforcing the idea of early spread of tumor cells to distant organs. Fast, specific, and sensitive detection of CTCs may have potential to enhance diagnosis, treatment, and cancer monitoring. CTCs may additionally be exploited for genotypic and phenotypic abnormalities representative of the tumor *in situ*.

CTC detection in peripheral blood has been reported in a number of cancer types, such as lung (Zhang et al., 2014), metastatic breast (Riethdorf et al., 2007), prostate (Hu et al., 2013), colorectal cancer (Kuboki et al., 2013), and gastrointestinal and biliary cancers (Al Ustwani et al., 2012; Tsujiura et al., 2014). Many of these cancers are diagnosed at late stages, resulting in high rates of mortality. Advancements in CTC collection, enrichment, and characterization have led to increased interest in the clinical use of CTCs. Studies in various organ systems have consistently shown that CTCs rarely exist in the blood of healthy subjects, consolidating their utility in the clinical laboratory (Sastre et al., 2008; Hou et al., 2013; Tsai et al., 2016), while supporting the premise of CTCs with the potential of a powerful biomarker.

Various available CTC detection technologies have expanded their use from simply diagnostic markers to tools to evaluate overall survival, risk of metastasis, and response to therapy. Recently, using the Veridex Cell Search System, the only FDA approved method for CTC enumeration in whole blood, Weissenstein et al., found a strong correlation between median overall survival in metastatic breast cancer patients with <5 CTC/7.5 ml vs. those with ≥5 CTC (*p* = 0.00006; Weissenstein et al., 2012). In a review of multicenter study of 1,358 individuals, Miller et al. found a highly significant median overall survival in favorable CTC counts vs. patients with unfavorable CTC counts with metastatic breast cancer, metastatic colorectal cancer, and prostate cancer (*p* < 0.0001). Additionally, patients enrolled in therapies that decreased CTC counts displayed improvements in overall survival, pointing to the utility that CTC analysis in the response to anti-cancer treatments holds (Miller et al., 2010). Studies have also taken advantage of the current available methods to correlate tumor dissemination and stage to CTC count. Hiraiwa et al. showed that CTC counts were higher in metastatic patients than in non-metastatic esophageal, gastric, and colorectal cancers and were significantly correlated to advanced tumor stages. High CTC count, defined as 2 or more CTCs per 7.5 ml in this study, was linked to pleural and peritoneal dissemination (Hiraiwa et al., 2008). Further, validation in clinical settings will establish CTC detection as a marker for sensitive and non-invasive cancer diagnosis, treatment evaluation and prognosis.

## CTC enrichment and detection

CTCs are rare cells, detected in numbers ranging from 1 to 10 s per ml whole blood, among billions of red blood cells and millions of leukocytes. CTC detection and isolation remain being technologically challenging (Joosse and Pantel, 2013). In the infancy of this rapidly growing field, current CTC detection and analysis rely mainly on various methods of enrichment.

CTC enrichment approaches exploit the unique biological and/or physical properties of this specified tumor cell type among vast numbers of peripheral blood cells, in order to increase CTC recovery by many orders of magnitude. Immunoaffinity platforms select for CTCs based on the expression of specific surface antigens, through either positive or negative selection. Since most carcinomas express epithelial markers, epithelial cell adhesion molecule (EpCAM) is most commonly used in antibody-based positive selection with commercial technologies. Negative selection depletes other mononucleated cells through anti-CD45 antibody use. Established collection methods have employed these proteins to attract and adhere CTCs to columns, microposts, or magnetic apparatus (Alix-Panabières and Pantel, 2014).

Two early platforms, magnetic-activate cell sorting system (MACS), and Dynabeads use magnetic fields for attract CTCs to anti-EpCAM antibody coated magnetic microbeads (Nagrath et al., 2016). Similarly, CellSearch (Janssen Diagnostics) uses anti-EpCAM conjugated ferrofluid nano-particles to immunomagnetically capture CTCs, which may then be differentiated from contaminating leukocytes based on positive cytokeratin or EpCAM staining and negative CD45 staining (Hayes et al., 2006). Despite much progress in platform development, CellSearch remains the only FDA approved method of whole blood CTC enrichment and enumeration. Another platform, MagSweeper (Stanford University), uses magnetic rods, stirred through diluted blood samples, to attract CTCs pre-labeled with EpCAM-magnetic beads (Talasaz et al., 2009). This platform was one of the first to enrich CTCs with a notably higher purity than its predecessors. Importantly, it has the ability to isolate live CTCs without perturbing gene expression throughout the enrichment process, providing viable CTCs for analysis (Krebs et al., 2014).

Microfluidic devices, which allow separation of CTCs, from small fluid volumes under laminar flow, are promising technologies. Nearly 10 years ago, Nagrath et al. was able to selectively and efficiently isolate CTCs from whole blood of 115/116 (99%) cancer patients using anti-EpCAM-coated posts with this “CTC-Chip” platform, eliminating the need for pre-labeling or sample processing (Nagrath et al., 2007). New microfluidic approaches have appeared since then, including methods taking advantage of physical properties as well, making the platform applicable to isolation of CTCs that lack or have down regulated EpCAM expression (Stott et al., 2010; Ozkumur et al., 2013).

Positive and negative CTC enrichment is also tested based on physical properties alone. Tumor cells and CTCs are generally thought to be larger (>8 μm) than hematologic cells (Vona et al., 2000, 2004; Hosokawa et al., 2013). Size-based filtration methods, such as isolation by size of epithelial tumor cells (ISET) through membrane filters with size exclusive pores, have been previously used to isolate individual CTCs and achieved higher sensitivity than CellSearch (Hofman et al., 2011; Hou et al., 2011).

As CTCs extravasate and intravastate the circulation, they undergo massive deformations in their structure due to mechanical forces which they endure. Cancer cells are known to be more deformable than normal cells, a quality that is correlated to their metastatic potential and is exploited by some enrichment platforms (Byun et al., 2013; Park et al., 2016). Additionally, platforms utilizing tumor cell property differences in electrical charge and density have been reported (Müller et al., 2005; Fabbri et al., 2013; Yoo et al., 2016).

While many of the platforms available have been able to detect CTCs in blood samples, these small peripheral blood collections, of several milliliters, may not be representative of the of the entire patient blood volume. Recently, an antibody coated medical wire (CellCollector; Gilupi GmbH) capable of detecting EpCAM and cytokeratin positive CTCs *in vivo* was introduced. In a recent study, a 30 min incubation period, in which the wire was exposed to circulating blood in the arm vein of lung cancer patients, showed over a 2-fold increase in CTC detection in comparison to CellSearch (Gorges et al., 2016b). Further, efforts to enhance the biocompatibility of these wire coatings, have been employed to maximize functionality for downstream processes such as sequencing analyses of captured cells (Scherag et al., 2017).

Once enriched, CTCs are detected/confirmed through various techniques. Immunocytological and molecular approaches are the most commonly employed. Immunocytochemistry (ICC) may differentiate CTCs from contaminating cells through biomarker detection. Such biomarkers can be specific for nuclear content, epithelial proteins (i.e., cytokeratins), and hematopoietic markers (i.e., CD45). A common immunocytological CTC definition, currently used by CellSearch and other platforms, is a Nucleus^+^/CK^+^/CD45^−^ cell. However, it should be noted that CTC designation depending primarily on epithelial marker expression may lead to false negatives by failing to detect CTCs that have undergone EMT (Lustberg et al., 2012). As cell phenotypes can vary in different malignancies, the heterogeneity of CTCs pose barriers to efficient and thorough detection by liquid biopsies.

Reliance on epithelial markers, which most epithelial carcinomas express, for enrichment and identification, fails to capture subpopulations of CTCs, such as mesenchymal cells, that may harbor clinically important information. Currently, cancer studies in breast and prostate have already demonstrated that mesenchymal marker expression by CTCs is associated with poorer survival (Aktas et al., 2009; Yokobori et al., 2013). Recently, negative depletion strategies that enrich CTCs in phenotype-independent ways have been introduced in an effort to solve this problem and enhance detection. Immunostaining of CD45 and cell sorting with flow cytometry was used to enrich the breast cancer CTC population (Lara et al., 2004). Multi-marker Immunomagnetic Negative Depletion Enrichment of CTCs (MINDEC), relies on depletion of non-CTCs as opposed to targeting specific properties of CTCs (Lapin et al., 2016). This technique is based on a multi-marker antibody cocktail (CD45, CD16, CD19, CD163, and CD235a/GYPA) to target various contaminating blood classes. This technique has shown high enrichment efficiency of both epithelial and mesenchymal CTCs, with better hematopoietic depletion than CD45 alone. Additional, novel cell surface marker-independent techniques have been shown to effectively detect CTCs in epithelial and non-epithelial malignancies in the absence of cell surface tumor markers. For example, a novel method introduced by Zhang et al., selectively labeled CTCs through GFP expression in human samples and cancer cell lines transfected with tumor selective replicating HSV-1 with a high detection efficiency (Zhang et al., 2016).

Table 1 shows various CTC isolation methods used in the last few years.

**Table 1:** CTC isolation techniques.

**CTC isolation method**	**Disease**	**Comment**	**References**
Size-based filtration method: Use of membrane filters with size exclusive pores	Multiple carcinomas	Isolation of CTCs based on cell size to isolate them from leukocytes	Vona et al., 2000, 2004; Hosokawa et al., 2013
Veridex cell search system: Anti-EpCAM antibody coated microbeads	Multiple carcinomas	The only FDA approved	Allard et al., 2004
Magnetic-activated cell sorting system (MACS)	Breast cancer	Enrichment of the CTC population	Lara et al., 2004
Use of electrical charges and density properties of cancer cells	Primary breast cancer	CTCs detected in 8.3% of patients before surgery. After Chemotherapy, CTCs detected in 44% of previously negative patients	Müller et al., 2005
Cell Search System	Gastro-intestinal cancers	High CTC number corrected with metastasis and with low survival	Hiraiwa et al., 2008
MagSweeper: Magnetic rods	Metastatic breast cancer	Higher purity than the Cell search system	Talasaz et al., 2009
Microfluidic devices: Microfluid approaches taking advantage of physical properties	Metastatic prostate cancer	Isolate CTC with down regulated EpCAM	Stott et al., 2010; Ozkumur et al., 2013
Combination of epithelial and mesenchymal markers: Use combination of antibodies to select CTC with epithelial and mesenchymal properties	Localized and metastatic colorectal cancer	Avoid CTC phenotype problem	Deneve et al., 2013
MINDEC: Multi-Marker Immuno-magnetic Negative Depletion Enrichment	Metastatic pancreatic cancer	Enhanced negative depletion strategy—MINDEC-based on multi-marker (CD45, CD16, CD19, CD163, and CD235a/GYPA) depletion of blood cells	Lapin et al., 2016
GILUPI cell collector: cell Collector-Wire coating: EpCAM and Cytokeratin antibody coated medical wire	Lung cancer	Isolation of CTCs from peripheral blood to overcome the blood volume limitations	Gorges et al., 2016b
Wire coating enhanced: Enhanced wire coting to maximize functionality	Breast cancer cells	Improvement of the wire technology	Scherag et al., 2017

Newer methods have employed combinations of epithelial, mesenchymal, tumor-specific, and tissue-specific marker expression (Pantel and Alix-Panabieres, 2013).

Additionally, nucleic acid-based technologies have provided an alternate avenue (Yu et al., 2011), as improvements in non-fixating enrichment procedures have allowed for the use of RT-PCR and qRT-PCR to amplify single or multiple gene transcripts for CTC detection.

Most recently, emerging single-cell sequencing techniques have shifted the field toward individual CTC analysis of genetic alterations associated with tumor mechanisms, clinical outcomes, therapy response, and drug targets and resistance. The usefulness of genomic analyses, however, is limited by heterogeneity between cancer subtypes, presenting barriers toward finding universal markers. Similarly, all the current enrichment, detection, and analysis techniques available harbor their own technical challenges and limitations. Most of these are outside the scope of this discussion.

## CTCs in pancreatic cancer diagnosis

Pancreatic cancer is one of the deadliest malignancies. Pancreatic ductal carcinoma (PDAC) makes up the majority of pancreatic cancers. While advancements in the treatment of other cancer types may have led to significant improvement in patient survival, advancements in pancreatic cancer research have not been met with the same success. PDAC incidence has remained stable over the last 30 years and the lack of fruitful therapies and new/useful diagnostic methods have yet to be changed in pancreatic cancer (Ryan et al., 2014). With current therapies improving survival outcomes by only a few months, pancreatic cancer patients face a 5 year survival rate of only 7%. Gemcitabine, the first-line of PDAC therapy, only modestly improves survival in advanced pancreatic cancer, while the clinical benefit of combinational-targeted therapies (Erlotinib+Gemcitabine) has proven to have only slight benefit, increasing overall survival by less than a month (Burris et al., 1997; Moore et al., 2007). Recent work in combinational chemotherapeutics has led to a promising approach, FOLFIRINOX (Oxaliplatin, Irinotecan, Leucovorin, and 5-fluorouracil), which has almost doubled survival in metastatic pancreatic adenocarcinoma patients to 11.1 months compared to 6.8 months with single-agent gemcitabine (Conroy et al., 2011). In another promising approach, albumin bound paclitaxel (Abraxane) plus standard gemcitabine therapy increased overall survival to 8.5 months compared to standard gemcitabine therapy with an overall survival of 6.7 months (Von Hoff et al., 2013). The toxicity associated with these regimens is unfavorable and should be used in patients with good performance status. Unfortunately, 5 year survival rates remain relatively unchanged. It is estimated that by 2030 pancreatic cancer will be the second leading cause of cancer-related deaths (Rahib et al., 2014; Dawson and Fernandez-Zapico, 2016).

The idea of a liquid biopsy, which could reveal diagnostic and prognostic information about a patient's state, has been gaining much traction in the past 10 years. In one of the earliest studies, with 12 types of metastatic carcinomas in 964 patients, CTCs were successfully detected in patients with pancreatic cancer using CellSearch, albeit in lower numbers than the other cancers (Allard et al., 2004). Below, we summarize the studies of CTCs in the diagnosis, staging, and prognosis for pancreatic cancer patients.

Pancreatic cancer is a fast progressive disease and its early diagnosis is challenging. Initial pancreatic cancer diagnosis depends largely upon symptoms, which would only appear late when tumor have fully progressed and are not specific to be recognized at early stages. Due to the pathobiology and aggressiveness of PDAC, by the time anorexia, early satiety, pain, and weight loss start present, the disease has already progressed, leaving little room for a favorable prognosis. Additionally, of the 15% of patients seeking medical care 6 months prior to diagnosis, 25% have symptoms resembling upper abdominal disease that may lead to misdiagnosis (DiMagno, 1999). Affirmative diagnosis is made by tissue biopsies obtained by surgery, image guided CT biopsy, or fine needle aspiration through endoscopic ultrasound (EUS-FNA). Despite its widespread use, EUS-FNA does have diagnostic drawbacks, specifically a sensitivity range from 75 to 94% and a specificity of 78 to 95%, with low but lethal complications such as pancreatitis and bowel perforation (Court et al., 2015; Bournet et al., 2016). For CTC detection to be adopted to pancreatic cancer diagnosis, it must be useful for early diagnosis and/or monitoring treatment responses. A key performance milestone necessary for the implementation of CTC technologies is an understanding of disease stage at which CTCs can be detected. At the same time, it should be kept in mind that CTC research in pancreatic cancer is at nascent stage, while CTC detection methods and criteria vary largely between studies. It is important to critically analyze the markers available and to characterize CTCs at different stages of cancer progression.

CTC detection has been explored in early diagnosis of various cancers and CTCs have been detected prior to tumor detection by traditional methods. A recent study, for instance, found that CTCs could be detected 1–4 years before lung cancer became detectable through CT-scan screening in the same patients (Ilie et al., 2014). For breast cancer, the American Society of Clinical Oncology (ASCO) has already approved the use of CTCs as a tumor marker, creating new directions for early breast cancer diagnosis (Harris et al., 2007).

Similar results have been obtained in pancreatic cancer. In a mouse model of PDAC, Rhim et al. found that inflicted pancreatic cells underwent EMT early during cancer development. These cells with EMT were predicted to represent early cancer cells, as the extent of EMT correlated well to invasive properties in tumor cells, facilitated their intravasation to circulating the blood and hepatic seeding, prior to the manifestation of primary tumors (Rhim et al., 2012). Additionally, blood samples from patients with pancreatic cystic lesions were detected to contain pancreas epithelial cells, at a time prior to cancer diagnosis. These findings suggested that pancreatic cell appearance in circulating blood precedes *in situ* tumor formation, and detection of CTCs could be an early biomarker for PDAC early diagnosis.

CTC-specific gene expression has been explored as surrogate markers for early cancer detection. Such studies mostly detect CTCs via detecting their expression of epithelial proteins. Reverse transcription-coupled polymerase chain reaction (RT-PCR) could be used to examine potential epithelial markers in CTCs derived from tumors of the epithelia. Soeth et al., evaluated cytokeratin 20 (CK20) detection, through RT-PCR detection from the marrow and venous blood in pancreatic ductal carcinoma patients. CK-20 positivity was detected in cells of 52 of 154 patients in venous blood, where higher CK20 level was correlated to UICC-tumor stage (Soeth et al., 2005). In a study of 34 pancreatic cancer patients prior to treatments, de Albuquerque et al used immunomagnetic enrichment for CTCs in peripheral blood based on mucin-1 and EpCAM expression. Subsequently, multi-marker RT-PCR analysis was used to detect tumor-associated transcripts, including KRT19, MUC1, EpCAM, CEACAM5, and BIRC5. CTCs with at least one marker in peripheral blood were detected in 47.1% patients prior to undergoing treatment (de Albuquerque et al., 2012). Detection efficacy was increased with the use of multiple markers as opposed to single marker use, indicating differential gene expression among CTCs from the same cancer patient.

Zhang et al., used an alternative strategy by enriching and identifying CTCs through a combination of CD45 and CK with a FISH-CEP8 probe in 22 pancreatic cancer patients. CTCs were detected from 15 of the patients, with CTCs ranging from 0 to 60 cells/3.75 ml of blood. In comparison, healthy controls, and patients with benign pancreatic tumors were negative for detection of CTCs, and sensitivity and specificity of CTC detection in pancreatic diagnosis were determined to be 68.18 and 94.87%, respectively, when using 2 cells/3.75 ml as cutoff. CTC-positive patients exhibited metastasis and poorer survival rates upon a 1.5 year follow-up. CTC positivity did not correlate significantly to CA19-9 levels of the *in situ* tumor. It is well-known that, though CA19-9 has a high sensitivity and specificity in advanced pancreatic cancers, its diagnostic usefulness is questionable to diseases at early asymptomatic stages. The combination of CA19-9 and CTC positivity in the study above increased detection rates from 68.18 to 77.3% (Zhang et al., 2015). The results from this study suggest CTCs as biomarkers for the diagnosis of early stage pancreatic cancers, in asymptomatic patients, and from patients with normal CA19-9 plasma levels. A later study by Xu et al., used a similar approach in 40 patients and dramatically high detection rates in PC patients (90%). Diagnostic rates increased to 97% when combining CTC ≥ 2 and CA19-9 > 37 μmol/L as a cutoff. Identification of chromosomal instability in CTCs, characterized as chromosome 8 triploids, showed a significantly statistic prognostic correlation. Patients with triploid CTCs < 3 displayed both higher 1 year and overall survival compared to those with ≥ 3 (Xu et al., 2017).

Doublecortin-like kinase 1 (DCLK1) may be another marker for CTC detection in early PDAC stages. Prior work suggests that Dclk1 marks stem cells, being able to differentiate cancer from normal stem cells. Interestingly, this marker protein is overexpressed in pancreatic and colorectal cancers (Nakanishi et al., 2012; Bailey et al., 2014). Qu et al., found elevated serum DCLK1 levels in stage I and II PDAC patients relative to controls and a decline in stage III and IV patients to levels similar to those seen in control patients. Diagnostic utility analysis showed both DLCK1 sensitivity and specificity to be significant in stage I and II patients. Furthermore, the investigators evaluated DCLK1 in the KPC mouse model, finding the serum DCLK1 levels to be significantly elevated as early as 5 weeks in KPC mice, compared to control mice. Over 50% of CTCs isolated from KPC mice whole blood were DLCK1^+^, suggesting a possible biomarker to be used in conjunction with CTCs for detection of early stage pancreatic cancer (Qu et al., 2015).

Kulemann et al., investigated the usefulness of CTC detection in pancreatic cancers from both localized and advanced stages. Peripheral blood from PDAC patients was used to capture CTCs for cytological and KRAS mutational analysis using the ScreenCell isolation method (Kulemann et al., 2015). High CTC detection efficiency as low as 2 cells/ml was calibrated by spiking experiments with healthy donor blood. CTC *KRAS* mutations were identified in 8 of 11 PDAC patients (73%). This is in sharp contrast to conventional biopsy-based diagnosis for the same patients, by which 2 of 11 samples (18%) were cytologically categorized as negative/non-diagnostic, while the rest exhibited abnormal morphology (18%) or were categorized as suspicious (64%). Moreover, the authors found no difference in the detection rate between early and advanced diseases, suggesting that CTCs are disseminated from primary tumors early in disease development and can be used to diagnose pancreatic cancer at initial stages where curative surgery may be available. It should be pointed out that this finding is contrary to those shown by Soeth et al. (2005), in which significant stage dependent differences were observed in CTC detection. Further studies are needed to clarify whether the difference is due to the differences in CTC detection strategies.

## CTCs in metastasis and early cancer

Early local invasion and metastasis are prominent factors in the poor prognosis of pancreatic cancer, as most patients are found with metastatic disease at diagnosis (DiMagno et al., 1999; Pandol et al., 2009). The impact of CTCs on pancreatic cancer metastasis, recurrence, and prognosis has been investigated.

A recent 9-cohort meta-analysis of separate studies using CellSearch and RT-PCR detection methods, involving 623 pancreatic cancer patients altogether, revealed associations between CTC detection and poor prognosis. Out of 623 patients, 268 (43%) were classified as CTC positive and displayed poor progression-free survival and worse overall survival than those in the non-CTC group (Han et al., 2014).

Using the CellSearch enrichment method, Kurihara et al., investigated the utility of CTCs in peripheral blood as a marker of clinical outcomes in 26 patients with pancreatic cancer. CTC positivity was found in 11 of 26 pancreatic cancer patients (42%). The authors demonstrated a significant difference in median survival times between CTC positive and negative patients, for 110.5 and 375.8 days, respectively (*P* < 0.001; Kurihara et al., 2008). Given that the detection method was shown to have 100% specificity, with no CTCs detected in the non-cancer groups, CellSearch detection strategy may not be sensitive enough, as no CTCs were detected from the other 58% pancreatic cancer patients. These results suggest the need of developing more sensitive methods to detect positive CTCs in all pancreatic cancer cases.

Similarly, de Albuquerque et al found a correlation between CTC positivity (47% of patients) and median progression-free survival (PFS). Patients with at least one tumor-associated transcript found in their CTCs, enriched from peripheral blood using immunomagnetic EpCAM and mucin 1 detection, had a PFS of 66.0 vs. 138.0 days in those who did not. Intriguingly, CTC enumeration was found to have no correlation to clinicopathological features of the disease, including metastasis status and tumor stages (de Albuquerque et al., 2012).

Bidard et al. studied CTC detection rates in a subset of 79 patients with locally advanced pancreatic carcinoma enrolled in the LAP 07 trial. The primary study (LAP 07) assessed the effect of subsequent chemotherapy vs. chemo-radiotherapy continuation on overall survival in patients whose disease was controlled after 4 months of chemotherapy alone. The patient subgroup was screened for CTCs using CellSearch technology prior to chemotherapy administration and 2 months after treatment. While the investigators found that CTC positivity was not prognostic of PFS, they found it to be an independent prognostic factor associated with poor tumor differentiation and shorter overall survival (Bidard et al., 2013).

Bissolati et al., used the same CellSearch technique with systemic and portal vein blood of 20 patients undergoing pancreatic resection. No significant differences in both overall survival and disease-free survival between CTC-positive and -negative groups. The authors did, however, find a higher incidence of liver metastasis upon a 2 and 3 year follow up in the CTC-positive portal vein group (Bissolati et al., 2015).

Similarly, an early study investigated CTC positivity in 67 intraoperative patients with biliary-cancer. Molecular detection of CEA mRNA-positive CTCs from peripheral, central, and portal veins via RT-PCR was associated with a significant incidence of hematogenous metastases compared to CTC-negative patients (37.5 vs. 11.4%; Uchikura et al., 2002). Nonetheless, whether surgical resection of the pancreas itself may contribute to tumor cell shedding remains to be addressed. Pancreaticoduodenectomy (PD), involving the pancreatic head, and distal pancreatosplenectomy (DPS), involving the pancreatic body and tail, are standard surgical procedures. Both require necessary mobilization of the pancreas, and may lead to CTC dissemination via the portal vein to increase the risk of liver metastasis (Kuroki and Eguchi, 2017).

Table 2 represents data showing association between CTC presence and pancreatic cancer stage and outcome.

**Table 2:** Association between CTC presence and disease stage and outcome.

**References**	**Finding**	**Number of patients**	**CTC Detection method in pancreatic cancer patients**
Allard et al., 2004	First detection of CTC in pancreatic cancer biopsies	16	Cell search
Soeth et al., 2005	Higher CK20 in the blood correlated with tumor stage	154	CK20 RT-PCR
Kurihara et al., 2008	Association between presence of CTC and survival	26	Cell Search
de Albuquerque et al., 2012	Association between presence of CTC and decreased PF survival. No association between presence of CTC and disease stage	34	Multi marker RT-PCR
Bidard et al., 2013	No PF survival association but association with poorer overall survival	79	Cell Search
Han et al., 2014	Association between CTC detection and poor prognosis	623	Cell Search and RT-PCR (9-cohort meta-analysis study)
Zhang et al., 2015	CT-positive patients exhibited metastasis and poorer survival	22	CD45 and CK with FISH-CEP8 probe
Xu et al., 2017	Diagnostic rate increased to 97% when combining CTC>2 and CA19-9>37	40	NE-iFISH
Kulemann et al., 2015	No correlation between CTC and stage. CTC could be used as early marker	11	Screen Cell Isolation Method
Bissolati et al., 2015	No PF or overall survival association. But higher liver metastasis incidence	20	Cell Search

Chausovsky et al., used RT-PCR to examine the usefulness of CK20 expression in CTCs in the diagnosis of metastatic lung, stomach, colon, and pancreatic cancers (Chausovsky et al., 1999), since CK20 has been shown to not be transcribed in cells of hematopoietic lineage (Burchill et al., 1995). Chausovsky concluded that CK20 is a potential biomarker for detecting metastasis, with a sensitivity of 22/28 (78.6%) in patients with metastatic pancreatic cancer. Cytokeratins are used conventionally to characterize cancer cells of epithelial origin (Cooper et al., 1985; Lane and Alexander, 1990). Combination of cytokeratin and additional gene expression may improve the efficacy of CTC detection.

Poruk et al., assessed the potential of CTCs as biomarkers in 50 patients prior to surgical resection, based on EMT-related epithelial and mesenchymal marker expression. CTCs were acquired from blood samples through the method of ISET. CTCs were further identified by immunofluorescence staining with antibodies against pan-cytokeratins and the mesenchymal cell protein, vimentin. This analysis found that 78% of patients had CTCs expressing cytokeratin and 67% co-expressed vimentin, while no CTCs were found to express vimentin only. The authors found a significant association between cytokeratin only positive CTCs and worse survival. Interestingly, co-expression of vimentin was predictive of recurrence (*p* = 0.01). Of the patients diagnosed with metastatic cancer at the time of surgery, all the CTCs were positive for dual staining (Poruk et al., 2016). These findings indicate the involvement of EMT mechanism in metastatic progression. EMT would render CTCs heterogeneous and multi-marker analysis would have to be employed in order to ensure a comprehensive detection of all CTCs in a patient blood sample.

A recent study enumerated CTCs independently of surface marker status using a GFP expressed tumor selective Herpes Simplex Virus replicated based on telomerase activity. Transfected cells of 290 samples of patients with different solid tumors were examined and CTCs were detected in patients with epithelial and non-epithelial tumors from as little as 4 ml of blood. PC patients had a positive CTC detection rate of 88.2% across various stages and had the highest average number of cells identified per samples (43.1). Additionally, CTC detection rates increased to 100% in PC patients with regional lymph node metastasis but no distant metastasis (N+M0), further supporting the use of CTCs as a biomarker in disease progression (Zhang et al., 2016). Other recent phenotypic-independent enrichment platforms have shown some success in CTC enumeration regardless of epithelial or mesenchymal surface proteins. Negative selection of hematopoietic cells in blood samples of PC patients using MINDEC showed a CTC detection rate of 71%. Further, characterization of the enriched cells showed the presence of both epithelial and mesenchymal CTC populations. While the high rate of positivity in this proof-of-principle study, in comparison to previous phenotype specific platforms, could be due to the authors use of patients with metastatic disease only, its ability to detect both epithelial and mesenchymal cells, in addition to CTC clusters, marks a progressive trend toward comprehensive detection of both epithelial and mesenchymal CTCs with one technique (Lapin et al., 2016). More importantly, both CTC surface marker-independent enrichment techniques allow for the viability of collected cells to be subsequently used for downstream genetic analysis without compromise from high background leukocyte levels.

Recent works characterizing EMT found CTCs positive for both epithelial and mesenchymal markers in peripheral blood of breast cancer patients (Yu et al., 2013). Studies in mouse models have provided insight into the composition of CTCs in pancreatic cancer. Single-cell RNA sequencing revealed the expression of both epithelial and mesenchymal markers in KPC *LSL-KrasG12D, Trp53flox/flox or* +, *Pdx1-Cre* (KPC) mouse pancreatic tumors. Moreover, the authors observed substantial loss of the classical *E-cadherin* expression, suggesting that some CTCs of epithelial lineage could indeed adopt a partial mesenchymal stromal phenotype through EMT, while retaining other epithelial features such as cytokeratin expression (Ting et al., 2014).

Different from epithelial cells, most mesenchymal stromal cells harbor certain stem cell properties, being able to be induced to differentiate into more mature cells (Zhau et al., 2011). The EMT phenotype is usually associated with expression of cancer stemness markers (Kong et al., 2011). Compared to other cancers, however, very little is known about stemness in pancreatic cancer CTCs.

In breast cancer, expression of cancer stem cell markers in CTCs is a sign of increased the metastatic ability (Papadaki et al., 2014). The expression of cancer stemness marker ALDH1 on CTCs was found to correlate to the stage of the disease and to the expression of EMT markers vimentin and fibronectin in prostate cancer patients (Raimondi et al., 2011). A study by Barrière et al. (2012) aimed at the detection of CTCs endowed with mesenchymal and/or stem cell characteristics, at the time of initial diagnosis with breast cancer, found that EMT and cancer stemness occur in the primary tumors and are associated with an enhanced ability for tumor cells to intravasate in the early phase of cancer development.

Multiplex transcriptome profiling of single CTCs revealed presence of sub-populations of CTCs expressing multiple pro-cancer transcripts including cancer stem cell markers such as CD44 and CD24 (Gorges et al., 2016a). So far multiple markers have been used to detect CTC stem cell properties in CTCs, including CD44, CD133, CXCR4, ABCG2, and ALDH1. Other markers used uniquely for pancreatic cancer CTCs include CD24 and c-Met (Yang et al., 2015). Whether CTCs with mesenchymal or stem cell characteristics may be used as a marker for aggressiveness of the disease remains to be evaluated in future studies.

## Challenges

There has been much progresses over the last decade to overcome the initial barriers of CTC research in the laboratory and clinic. Significant technological development has been made for CTC detection, enrichment, and molecular characterization. On the other hand, CTC research in gastrointestinal cancers had a late start relative to other human malignancies (Allard et al., 2004). Due to gastrointestinal biology, pancreatic cancer detection via CTCs has its own set of challenges. It is widely proposed that the liver sequesters CTCs as they pass into the systemic circulation via the portal vein (Jiao et al., 2009). The predominant dissemination of pancreatic cancers to the liver have supported this notion. A recent report detected CTCs in 100% (14/14) of portal vein samples of patients with pancreaticobiliary cancers as opposed to under 25% (3/14) when peripheral samples were used for detection (Catenacci et al., 2015), a result consistent with the notion that higher CTC numbers are detected in portal vein as opposed to peripheral blood (Waxman et al., 2014). Furthermore, there was a significant increase in CTC detection in portal blood vs. peripheral blood, with a mean of 125.64 CTCs/7.5 ml as opposed to 0.8 CTCs/7.5 ml (*p* = 0.01; Catenacci et al., 2015). This study further emphasizes the importance that the collection site plays in CTC detection.

While preliminary studies in various cancers are demonstrating the potential of CTCs in early cancer detection, the continuous data coming out using different platforms (exploiting size, density, charge, surface antigens, etc.) make it challenging to reach a consensus for clinical application. The diversity of methods for CTC enumeration and characterization can confuse the research and the clinical communities. There is a lack of large studies comparing enrichment and detection between CTC detection platforms. A pilot comparative study of 54 pancreatic cancer patients investigated differences between CellSearch and a marker-independent ISET CTC isolation (Khoja et al., 2012). The authors detected significantly more CTCs using ISET in comparison to CellSearch (93 vs. 40%). Similar studies in other cancers have pointed out the discrepancies between platforms (Farace et al., 2011; Hofman et al., 2011).

Discrepancies can partially be attributed to different types of carcinomas and their expression of surface markers. Furthermore, the differences in specificity and sensitivity may lead investigators to adapt different platforms for their specific study. This raises many concerns in regards to the identities of CTCs. As EMT facilitates CTCs with increased capacity for detachment and invasion, the loss of epithelial lineage marker expression makes the identification particularly difficult. We must further explore the limitations that certain platforms create in capture efficiency. For example, EpCAM expression, which CellSearch exploits, is heterogeneous and cleavage has been reported (Maetzel et al., 2009). Limitations of other epithelial cell markers have also been reported, such as the down regulation of CK20 in tumors leading to false-negatives (Vlems et al., 2002; Krebs et al., 2014). It would be ideal for platforms to detect both mesenchymal and epithelial characteristics of CTCs, and the platforms must be carefully validated.

Additionally, not only do detection rates vary by platforms, but also between cancers. There is currently no consensus on the cutoff value for CTC positivity, even within a single platform. Stringent parameters should be set for CTC use not only in detection, but also as a prognostic marker of clinical outcomes in pancreatic cancer. Although it may be difficult due to the differences in enrichment and detection between platforms, standardization across single or multiple platforms is paramount for future incorporation into the field.

Due to the complex nature of the metastatic process, disseminated cells may be clinically silent for long durations. In breast cancer, cytological assessment suggests that CTCs actively undergoing mitosis are most common in late-stage disease and have prognostic value (Adams et al., 2016). One study found that aberrant VCAM1 expression, a common complication of breast cancer, was crucial for the transition from dormancy to overt metastasis (Lu et al., 2011). We must continue to explore ways to stratify CTCs in ways that will allow us to distinguish indolent micrometastasis from aggressive CTCs prior to clinically significant metastasis in pancreatic cancer patients.

## Conclusion and future directions

Literature evaluating the diagnostic and prognostic role of CTCs in cancer is continuously being reported. Many studies in different malignancies have shown clear associations of CTCs with clinical cancer progression. Much of the current research is now shifting to CTC characterization in order to select appropriate therapies for individuals based on the gene signatures of the CTCs and to measure response to therapies. For example, CTC count now outperforms traditional response evaluation methods in patients with metastatic castration-resistant prostate cancer (Onstenk et al., 2016). With reports estimating the half-life of CTCs to be on the order of hours, their detection can provide a current representation of the malignancy (Meng et al., 2004; Stott et al., 2010).

Future investigations should thoroughly explore CTC response to pancreatic cancer treatments. Furthermore, *ex vivo* CTC culture and expansion experiments can improve our understanding of the mechanisms of dissemination and escape from dormancy. Single-cell sequencing with next-generation sequencing platforms is paving the way toward understanding the genetic makeup of CTCs and the clinical significance of their genomic alterations (Alix-Panabières and Pantel, 2014). A recent study in a pancreatic cancer mouse model used single-molecule RNA sequencing of CTCs to identify Wnt2 as an up-regulated gene in pancreatic cancer CTCs, which is implicated in cell-death suppression and cancer dissemination. In addition, the authors observed the same Wnt2 signaling aberrations in CTCs of 5/11 patients with metastatic pancreatic cancer (Yu et al., 2013). Such studies have the potential to improve our current clinical management, especially ones exploring new drug targets involved in cancer spread. CTC use as a biomarker is currently being investigated in over 360 open clinical trials registered on ClinicalTrials.gov (Alix-Panabières and Pantel, 2014).

Considering that methods have been developed that have the possibility of being used in the diagnosis, stratification of patients and monitoring of therapy, next efforts require a focus on validation of leading methods for aiding clinical care. For the methods chosen, the validation of the method for certification in clinical use followed by well-designed studies to show utility of the method in the clinical setting are necessary for approval of test for clinical application.

There are challenges in the pancreatic cancer field for development of a test that has utility in early diagnosis or choice of chemotherapy. For example, in the area of early diagnosis, a population at increased risk is needed to show performance of the method in detecting pancreatic cancer at an earlier stage than that achieved with current approaches. Currently, the Consortium on Chronic Pancreatitis, Diabetes, and Pancreatic Cancer (CPDPC) in the United States is developing a protocol for this purpose choosing patients with diabetes after age 50 as the best high risk group to use to identify early diagnostic biomarkers (http://cpdpc.mdanderson.org). Because about 1% of these patients will be found to have pancreatic cancer over 3 years after the diagnosis of diabetes, the CPDPC has determined that the study will require enrollment of 10,000 subjects. At present, the inclusion of measurement of CTCs is not being considered because of the technical difficulties involve with CTC measurements. On the other hand, once a proteomic, ctDNA and/or RNA technique is developed to identify patients with early pancreatic cancer, measurements of CTCs can be applied to this group for further characterization including choice of therapy.

Similarly, it is difficult to apply CTC technology to the choice of chemotherapy as the current therapies do not have a significant effect on long term survival. One the other hand, surgery does have significant effects on long term survival in a substantial percentage of patients. Thus, it seems that currently the best situation to develop a validated test for CTC measurements uses patients who are candidates for curative surgery. Hypotheses to be tested should focus on the role of CTC measurements in predicting the outcome of curative surgery and early demonstration of disease recurrence. Certainly, studies that show performance of CTC measurements in determining and monitoring outcome in surgical patients will have important impacts in disease management and are much more feasible than studies designed for early diagnosis.

Another area of significant importance in the field is the determination of the biology of CTCs. As these cells represent the metastatic process which is the key determinant of poor outcome in pancreatic cancer patients, a better understanding of the biology of these cells will be central to advancing our treatments. Are there unique mechanisms in pancreatic cancer that account for its high rate of metastasis? Are there properties of pancreatic cancer CTCs that account for its resistance to therapy? Exploring these questions will require advancing the methods of isolation and propagating these cells so that the biologic experiments including observing their behavior in cell culture and animal models can be performed.

## Author contributions

SP provided oversight and direction for the development of the manuscript. MP did literature searches and drafting of a primary manuscript. ME and RW provided additions and editing for the manuscript.

### Conflict of interest statement

The authors declare that the research was conducted in the absence of any commercial or financial relationships that could be construed as a potential conflict of interest. The reviewer RJ and handling Editor declared their shared affiliation, and the handling Editor states that the process nevertheless met the standards of a fair and objective review.
